# Pruritus in patients with amyopathic dermatomyositis

**DOI:** 10.1186/s13223-019-0334-5

**Published:** 2019-03-27

**Authors:** Dimitri Poddighe, Zaure Mukusheva, Maikesh Assylbekova

**Affiliations:** 1 Department of Medicine, Nazarbayev University School of Medicine, Kerei-Zhanibek Str. 5/1, Astana, 010000 Kazakhstan; 2 Department of Pediatric Rheumatology, National Research Center of Mother and Child Health, University Medical Center, Astana, Kazakhstan

**Keywords:** Amyopathic dermatomyositis, Pruritus, Omalizumab

## Abstract

Amyopathic dermatomyositis has been associated with the exposure to several drugs: the article by Jeimy et al. described the onset of this uncommon disease in a patient treated with omalizumab. Paradoxically, this patient complained of an intense pruritus and this finding has been reported by several authors observing patients with amyopathic dermatomyositis.

To the Editor,

We read with great interest the case report by Jeimy et al. [1], describing the onset of amyopathic dermatomyositis (aDM) in an adult patient receiving omalizumab therapy for severe asthma. As highlighted by the authors, this is the first case report of DM associated with the biological anti-IgE therapy, namely omalizumab, which is currently approved to treat unresponsive chronic spontaneous urticaria as well [1, 2]. Paradoxically, this clinical case was characterized with an intense pruritic dermatitis and, beyond the description of such a peculiar drug-disease association, it provides the opportunity to focus the attention on some clinical features of the “amyopathic” variant of DM, which is often difficult to be diagnosed. Indeed, delays in diagnosis can be due to the lack of clear clinical characteristics and typical biochemical changes.

Previously, we described a case of amyopathic juvenile DM (JDM) and, interestingly, we also noticed that important “erythematous, intensely pruritic cutaneous eruption” unresponsive to several drugs, as described by Jeimy et al. [1]. Specifically, our patient received several courses of H_1_-antihistamines (e.g. cetirizine, levocetirizine, loratadine, oxatomide) without achieving the complete control of pruritus: indeed, she required intra-venous chlorphenamine several times and even received low-dose amitriptyline, as prescribed by the specialist in pain management. This clinical aspect has oriented our attention on pursuing a diagnosis of allergy for months, given that the typical clinical signs of JDM, such as heliotrope rash and Gottron’s papules, have appeared much later during the clinical course, namely 10–12 months after the symptom onset. Similarly, our patient displayed neither significant abnormalities of muscle enzymes, nor specific autoantibody, but she responded to the immunosuppressive therapy and, in particular, to hydroxychloroquine, as a maintenance and steroid-sparing drug [3].

Other authors have reported this very pruritic aspect of skin manifestations in aDM. Concha et al. reported this feature as a frequent finding in their article that revisited the current diagnostic criteria for aDM. Indeed, these authors highlighted that pruritus has been considered in several classification criteria for aDM (e.g. Sontheimer 1997, Dalakas 2003) and it is more pronounced in this rheumatic disease rather than others (e.g. systemic lupus erythematosus) [4–6].

Recently, Galimberti et al. also reported intense pruritus in 55% of their study cohort, including 44 patients diagnosed with aDM (who represented 18% of all their DM patients). Most of their patients (female/male ratio = 10:1) were 50–70 years old at aDM diagnosis [7], but our case demonstrated that aDM can arise as juvenile form as well [3]. Interestingly, 19 out 24 of their aDM patients (corresponding to > 75%) were not affected by any malignancy [7].

Therefore, patients with “amyopathic” DM/JDM can develop a dermatosis characterized with very intense itching, which may mislead the diagnosis toward allergy diseases for long time and, as a consequence, delay the correct diagnosis and therapy.

## Abbreviations

aDM: amyopathic dermatomyositis
DM: dermatomyositis
JDM: juvenile dermatomyositis

## References

[1] Jeimy S, Basharat P, Lovegrove F (2019). Dermatomyositis associated with omalizumab therapy for severe asthma: a case report. Allergy Asthma Clin Immunol.

[2] Poddighe D, De Amici M, Marseglia GL (2016). Spontaneous (autoimmune) chronic urticaria in children: current evidences, diagnostic pitfalls and therapeutic management. Recent Pat Inflamm Allergy Drug Discov.

[3] Poddighe D, Cavagna L, Brazzelli V, Bruni P, Marseglia GL (2014). A hyper-ferritinemia syndrome evolving in recurrent macrophage activation syndrome, as an onset of amyopathic juvenile dermatomyositis: a challenging clinical case in light of the current diagnostic criteria. Autoimmun Rev.

[4] Concha JSS, Tarazi M, Kushner CJ, Gaffney RG, Werth VP (2018). The diagnosis and classification of amyopathic dermatomyositis: a historical review and assessment of existing criteria. Br J Dermatol.

[5] Dalakas MC, Hohlfeld R (2003). Polymyositis and dermatomyositis. The Lancet.

[6] Sontheimer RD (2002). Dermatomyositis: an overview of recent progress with emphasis on dermatologic aspects. Dermatologic Clinics.

[7] Galimberti F, Li Y, Fernandez AP (2016). Clinically amyopathic dermatomyositis: clinical features, response to medications and malignancy-associated risk factors in a specific tertiary-care-centre cohort. Br J Dermatol.

